# Professional Secresy

**Published:** 1858

**Authors:** 


### Professional Secresy.

It is regarded as a nice point to determine how far medical men are required to go in promising and observing secresy when in the discharge of their professional duties. We think that no sound, moral, conscientious, or intelligent physician could find the least embarrassment in coming to the conclusion as to what, for the ends of public justice, should be divulged; and what, for the protection of individual character and standing, should be withheld.

When a conspiracy against the rights, property, or reputation of an individual is contemplated, or a criminal offence against the laws is conceived and projected, to carry out which it is necessary to secure the aid and counsel of a medical man, then the duty he owes to himself, to society and his profession, demand an immediate exposure after he has shown the criminality of the offence and expended his efforts in dissuading, unsuccessfully, the parties from further steps in their criminal intents and purposes.

There is no code of ethics, professional or moral, which should prevent him from acting, under such circumstances, promptly and decidedly. The profession is not that retreat into which the guilty should fly for concealment or protection, neither is it the apologist for crime, nor the defender of wrong. If it were thus to degrade and debase itself to unholy purposes, it would be necessary only, in order that the offender should escape the justice he merits, and a just punishment for his offences, first to seal the mouth of the mediical man by consulting him, and making a clean breast of his guilt. And yet, very often, for the ends of justice and the development of the most important facts in criminal prosecutions, the testimony of one medical man would be essential to conviction. But the professional seal is upon his lips, his tongue is paralysed by his ethics, and he is not compelled to reveal the most important facts.

Far, very far is this his duty, for he is under obligations to society and public justice, and these obligations are even more binding and weighty because of the position he holds in society, and the numerous opportunities of discovery. There are, however, certain facts, which come under his cognizance, and which he is bound by his vows when accepting his degrees, to keep sacredly within his own bosom, because no good could result to community if publicly known, in cases where the wrong is confined to the individual, and that, too, the result of the weakness of our erring natures. He who would divulge under such circumstances, is unworthy a place in the profession, should be hurled from the high place to which he has been elevated, and treated with contempt and scorn by all honorable men.

The city of New York has recently furnished an instance of complicity in crime, and the divulgence of the facts of a criminal act, in which two or three of the medical men of that metropolis bore a rather conspicuous and unenviable part.—The facts in the case were of so peculiar a nature, and so intimately connected with the circumstances of a bloody tragedy recently enacted there, that the public have been made aware of them through the secular prints, and yet there were some particulars which have not been made known in this manner for prudential reasons.

The murder of Dr. Burdell was followed, as every one knows, who has heard aught of the circumstances of his assassination, by the arrest of Mrs. Cunningham, who claimed to be his lawful wife. Whilst awaiting her trial she was incarcerated in the Tombs, where she declared herself to be *enciente* by Dr. Burdell, and her form gave color to her declarations. After her release she consulted one Dr. Uhl, promising at the time, a liberal fee. Dr. Catlin was also required to give his attention, because, as she said "he was in her power." Dr. Uhl had some conscientious scruples in relation to the act, as the lady frankly acknowledged to him that she was *not* pregnant. He immediately consulted with an Attorney of the Court who advised him to wink at the proceeding, and to favor the plot in order that she should be dealt with as the law provided in such cases. He did so and made arrangements for the procurement of a newly born child at the Hospital, which was marked duly by the Hospital physician with nitrate of silver under the arm-pits and behind the ears, so that the stains would not be seen by the guilty persons, and he also furnished a recent placenta. The deceit was further carried out by bringing the child to a house in one of the streets of the city not far distant from the residence of Mrs. Cunningham, who was then informed of the exact locality and which she visited with the view to reconnoitre, habited as a "Sister of Charity" with the usual hood and black gown. Notwithstanding this disguise, Dr. Uhl, who was expecting some one on that errand, very plainly recognized the person of Mrs. C., herself. Dr. U. immediately after repaired to the residence of Mrs. C., when Mrs. Burns, Mrs. C's sister was required to go for the child, to which she consented. Dr. U. returned in advance to the house where the infant was to be found, and after waiting a few minutes, the "Sister of Charity" again appeared, who was recognized as before, to be Mrs. C. in quest of her Hospital protege, a fact of which she was ignorant, as Dr. U. had led her to believe he mother to be an actual resident of that locality, and willing to part with her child. She proceeded to the room,

(after making a signal answer to a question put by Dr. U. as per previous arrangement,) in which the child was kept, where she found all the "pomp and circumstance" of a recent accouchment. A basket was on the floor at the feet of the nurse in whose lap lay the young infant, and upon a cot the "sick mother" as she supposed. She was asked if she came for the child, the signal (the exhibition of a white handkerchief) was the silent reply, and taking the babe she retired.

The Dr. now returned home and very soon a strange gentleman called upon him and required his immediate presence at the house of Mrs. C., as she was in labor. Upon his arrival he was shown into a darkened room where were Mrs. Burns (her sister) and Dr. Catlin. Dr. C. brought in the vessel in which was bullock's blood and a placenta, and here, extended upon the couch, was Mrs. C., who was *groaning* and *grunting* and suffering from "unusually severe after pains." No one, so far as we have learned, ever made a vaginal examination, to ascertain the presentation position, the rupture of the membranes, the progress of the labor, the extension, the support of the perinæum, the restitution, the severance of the cord, the subsequent contraction of the uterus, nor the expulsion of the placenta. All this had transpired prior to Dr. Uhl's arrival, but it become necessary still, that the sheets be stained in the blood of the bullock, whether done as a modification of the ancient sacrifices or not, we are left wholly ignorant.

Very soon certain officers arrived, accompanied by Dr. Montagnie, to congratulate (doubtless) the lady upon her safe delivery. The child had been washed, and dressed in a new and splendid suite, much more elegant than the plain equipments which were provided for it at the Hospital. Dr. M. enquired of Mrs. C. whose child that was, who replied "it is mine, whose should it be but Dr. Burdell's, for I am his widow?" But the Dr. found it to be his Hospital foundling by the marks which he had purposely put upon it, and by the peculiar kind of string he tied the umbilical cord with.—The woman, Burns, maintained that it was "Mrs. Burdell's *baby*," when the officers attempted to take it away from the arms of "*Mrs. Burdell* (*!*)" lest it might be destroyed by these guilty wretches.

Such, in brief, are the particular circumstances of this attempt at a wicked and guilty deception, for which we are indebted to the "Medical Gazette" of N. Y., which, after giving a particular narration of the facts, proceeds to discuss the propriety of the course pursued by Dr. Uhl, and to condemn him for winking at, and encouraging the deception and fraud. We entirely agree in opinion with the writer, that the proper course for Dr. Uhl to have pursued would have been to scout the proposition, to have denounced it as wicked, and threaten the parties with an exposure if carried out. There is, however, some extenuation in this case, for the public mind will form its own conclusions more intelligently as to her complicity in the murder of Burdell, although the fraud is not proof of the fact. Yet it is fair to conclude that she who could so successfully plan and carry out a scheme of fraud, and act out the parts so well, could and would, we think, contrive and aid in any crime however remorseless and bloody. It will, also, be useful as a precedent and a warning to the profession whose morals are at loose ends, how they proceed in future in aiding and abetting crime. In every case of whatever nature, the medical man, when consulted, should not only discourage, and discountenance, but he is called upon to threaten the parties with exposure if they persisted, and this we think should have been the course which Dr. Uhl ought to have adopted.

In the same Journal above referred to, is the report of the arrest of a physician in Montreal, Canada, for producing criminal abortion, which crime is becoming quite too common and should be punished with due severity. We are now engaged in ferreting out some cases in which we have reason to believe aid has been furnished in the consummation of the wicked designs of those who resorted to it either for the purpose of concealing their shame, or for the equally criminal and unnatural motive of avoiding the pains of child-birth, or the cares of a family. Meredeth Reese, M. D., L. L. D., of New York, made a most able and interesting report to the A. M. A., at its late meeting, on the mortality of children, in which he exposes the wicked motives and conduct of those who commit fœticide for *any* purpose except to save the more valuable life of a mother. We are awaiting the publication of this report with much anxiety, that we may give to our readers such portions as will be most interesting and profitable, from time to time.

